# Editorial: Self and Memory: A Multidisciplinary Debate

**DOI:** 10.3389/fpsyg.2018.02676

**Published:** 2019-01-08

**Authors:** Rossella Guerini, Massimo Marraffa, Cristina Meini, Alfredo Paternoster

**Affiliations:** ^1^Department of Philosophy, Communication and Performing Arts, Roma Tre University, Rome, Italy; ^2^Department of Humanities, University of Eastern Piedmont, Vercelli, Italy; ^3^Department of Letters, Philosophy, Communication, University of Bergamo, Bergamo, Italy

**Keywords:** autobiographical memory, life narratives, phenomenological continuity, personal identity, post-traumatic stress disorders, psychotherapeutic vs. pharmacological treatments, semantic continuity, sense of self

## Background

There is no consensus about the definition of autobiographical memory and its contribution to our sense of self. Along the lines of Hoerl (2007), we organized the research topic by distinguishing three strands of theorizing which vary according to how they construe the relationship between autobiographical memory and the notions of episodic memory and self.

Autobiographical memory can be defined in terms of episodic memory, where the latter includes a reference to the self. As Tulving puts it, “episodic memory differs from other forms of memory in that its operations require a self. It is the self that engages in the mental activity that is referred to as mental time travel: there can be no travel without a traveler” (Tulving, 2005, pp. 14–15; emphasis added). Once this reference to a traveling self is built into the notion of episodic memory, a further step may be taken by construing such notion in terms of the phenomenological construct of prereflective self-consciousness. This path is taken, for example, by Prebble et al. (2013) who proposed a framework for the study of sense of self and autobiographical memory where an immediate and automatic sense of being the subject of experience (pre-reflective self-experience) is seen as the precondition for episodic autobiographical memory, which in turn—in virtue of its features of autonoetic consciousness and mental time travel—is a prerequisite for experiencing unity in our subjective experience of selfhood across time (phenomenological continuity). Thus episodic remembering makes it possible the unification of the self as a single entity that persists across time (diachronic unity) by conveying “the inherent ‘mineness' of the original experience into the present moment” (Prebble et al., 2013, pp. 818–819). In contrast, semantic continuity is a form of continuity that is not experiential but knowledge-based (the knowledge that the self exists over time), mediated by semantic autobiographical memory which enables us to construct life narratives.

Other models, however, view episodic memory as “only one possible aspect or instance” of autobiographical memory (Hoerl, 2007, p. 637, note 4). For example, Conway (2005) argues that episodic memories are experience-near sensory-perceptual-conceptual-affective summary features of short-time slices of experience, which are durably retained only if they have been embedded in autobiographical knowledge structures; otherwise, they are rapidly forgotten. Within what Conway terms “the self-memory system,” therefore, the notion of autobiographical memory is no longer defined in terms of episodic memory; only conceptual organization of episodic memories within the self-memory system transforms them into autobiographical memory and allows them to play a role in constructing and maintaining a coherent, stable mental representation of the self (the conceptual self) over time.

In this perspective, in contrast to Tulving's assumption that remembering past events serves to establish the sense of continuity of our self over time by virtue of a specific phenomenal quality (i.e., the immediate feeling that “I” experienced the remembered event), Conway proposes the opposite: it is the conceptual self (the present Me) that selects and also distorts personal memories so as to increase the sense of personal continuity. As a consequence, self-continuity is not “provided by the identity of the remembering I, but by the perceived similarity of the present and past Me” (Habermas and Köber, 2015, p. 153).

A third approach, well represented by Katherine Nelson's work, takes autobiographical memory as a subclass of episodic memories which involve a reference to the self, which is viewed however from a social interactionist standpoint (see, e.g., Nelson, 1996). The sense of self in time originates then from social-communicative interactions, and especially from sharing memory narrative which progressively lead children to rationalize memories of their experiences in autobiographical terms. Thus, Nelson meritoriously stresses more than other authors the role of relationships in constituting a sense of self, placing herself in the wake of an important and heterogeneous tradition going back (with different accents) to Vygotsky and, in the infant research and clinical domain, to the object relation and attachment theories.

On the backdrop of these three theoretical perspectives, the present research topic called interdisciplinary contributions concerning the relationship between autobiographical memory and the self, and notably their functional and dysfunctional interactions. What follows is an overview of the contributions that this research topic includes. It is divided into four sections with headers that describe the subthemes of the issue.

## Modeling the Link Between Autobiographical Memory and Sense Of Self

Tippett et al. build on their aforementioned model of the relationship between autobiographical memory and the self, making the hypothesis that diachronic unity (i.e., the belief that, despite changes, we are the same person across the lifespan) rests on phenomenological continuity (i.e., the experience of remembering the self over time during episodic recall). When phenomenological continuity is disrupted, however, diachronic unity can still be supported by semantic memory, which enables us to construct narratives from which semantic continuity emerges.

The hypothesis was tested in a study involving patients with mild-to-moderate probable Alzheimer's Disease (AD) and with amnestic mild cognitive impairment (aMCI), two conditions where episodic memory deficits disrupt phenomenological continuity. This alone should not lead to deterioration in diachronic unity, provided that semantic continuity is well-preserved; but if semantic continuity is also altered both in AD and aMCI, there should be an associated deterioration in diachronic unity. The latter was measured through a self-persistence interview (beliefs about self-persistence, explanations for stability/change); semantic continuity was assessed measuring autobiographical reasoning (connections between one's conceptual self and the events of one's life) and (temporal, thematic, and causal) coherence of narratives.

The findings in both aMCI and AD groups confirm the hypothesis that intact semantic continuity is sufficient to support diachronic unity. In the aMCI group, the quality of life narratives was largely preserved, as were measures of self-persistence, indicating that even when the continuity afforded by phenomenological re-experiencing is disrupted, semantic continuity is sufficient to support diachronic unity. However, the findings from the AD group indicate a more complex relationship between diachronic unity and semantic continuity than originally proposed in Prebble et al. (2013). For these findings reveal a divergence between two aspects of diachronic unity: the AD and aMCI groups did not differ from health controls in their beliefs about their persistence over time, but patients with AD showed deterioration in their ability to construct a sophisticated justification for their persistence across time. The authors suggest that in the AD group diminished life narratives that retain features of cultural life scripts are sufficient for strong subjective beliefs of self-persistence, but not for sophisticated explanations about persistence. Better semantic continuity, with the ability to weave high-quality life narratives, may scaffold the capacity to understand and explain one's diachronic unity, but this produces less certainty about their self-persistence.

Wheeler and Gabbert adopt Tulving's notion of episodic memory as a specialized subcategory of memory relating to the conscious recall of personally experienced events. Then they usefully emphasize the distinction between a self-generated cue and a self-referent cue—the former represents the critical properties of a target memory; the latter acts as a cue relating to an aspect of the self. On this background, they provide the first overview of the research work that showed that self-generated cues represent an effective and viable mnemonic technique which can aid recall in a variety of settings.

The authors also theoretically contribute to this research area by speculating on the memory mechanisms that lead to the improvement of memory through self-generated cues. Three mechanisms seem able to explain the effectiveness of self-generated cues. (1) Spreading activation models which view information in long-term memory as being represented by a network of associated concepts. It is possible to recall a given item from memory by recalling other information associated with the target; this is made possible through the process of activation spreading through the network. (2) The principles of encoding-specificity (or encoding-retrieval specificity), i.e., the idea that retrieval cues are effective only to the extent that information within the memory cue is also contained within the target memory trace created at the time of encoding. (3) Cue distinctiveness (or an absence of cue overload) which refers to whether a cue is uniquely associated with a target memory—if a cue is linked to multiple memory traces (and so is “overloaded”), then it becomes more difficult for that cue to activate the current target trace. The authors conclude by suggesting that self-generated cues represent a promising development in episodic memory domains.

Vanderveren et al. shift the focus to memory specificity and memory coherence, two characteristics of autobiographical memory that have been related to psychological well-being and the occurrence of psychopathology. To make a first attempt at a theoretical integration of memory specificity and memory coherence, the authors situate them within the Self-Memory System (SMS). Concerning memory specificity, people suffering from depression or PTSD are reported to experience specific difficulties retrieving episodes and tend to overgeneralise memories. Their semantically (rather than episodically) structured memory impedes time traveling and social life and, consequently, self-regulation—thus predisposing to further suffering. Nonetheless, the whole overgeneralisation literature can be traced back to Williams and Broadbent's Autobiographical Memory Test (AMT), which attested specific difficulties to retrieve personal memories cued by emotion words. In AMT episodic memories are circumscribed by time (no more than one day). Conversely, in SMS episodic memories are defined by event-specific information, which is a unity given by a continuity given by perceptual episodes not necessarily coincident with AMT's temporal slot; this discrepancy raises the problem of understanding what AMT actually measures.

Coherent memories are also associated to well-being. Coherence allows the construction of a unified meaning, which in turn promotes a sense of self-continuity. It is also a precondition for self-regulation, to the extent that it allows a subsumption of an event under a meaningful history. Nevertheless, some theoretical problems still remain unresolved, mostly concerning what relevant dimensions of coherence exactly are: temporal, causal, or thematic coherence? Or a mix of the three? Moreover, the prevalence of cross-sectional studies still leaves unanswered the developmental question: is memory incoherence a genuine predictor of psychopathology; or is rather one of its side effects?

The authors conclude by pointing out that the whole research on memory would beneficiate from a more unified view on specificity and coherence. More iterated models, such as SMS, do not consider the two as separate dimensions, but rather point out to their interconnectedness. Far from neglecting some peculiarities, such as episodic amnesia leaving largely preserving coherence, the authors still recommend further research from a more integrated prospective. With its emphasis on the interconnectedness between current personal goals (supervising coherence), episodic, perceptually vivid traces and long-term memories, SMS is in best position for this scientific new deal.

## Autobiographical Memory, Life Narratives, And The Construction Of Self-Identity

Marchetti et al. focus on adolescence in the development of an adult self, arguing that much more attention should be devoted to the role of body, against a tradition that puts at the forefront the integration of the psychic and the social. Indeed, according to the authors, the body is from birth (and even from the uterus) a constructive, and expressive vehicle of the self.

In order to understand how the body supports the construction of the identity in adolescence, the authors suggest using the method of analyzing what happens when something goes wrong, on the assumption that adolescents choose the body to express their discomfort, for instance, through self-injury. On this background, the authors' main thesis is that the capacity to mentalize represents a critical forerunner of the adolescent's perception of the physical self, insofar as the body is a carrier of implicit memories. Hence, troubles with mentalization in the child involve repercussions both on the child's behavior and the adolescent's psychic integrity. Violence against the physical self (in adolescence) could be an answer to the necessity of a change or an elimination of parts of a self- constructed in infancy and embedded in a bodily, implicit memory.

Finally, the authors point out the importance, particularly in adolescence, of taking into account one's own body in relation to others' bodies (and others' minds): relationships do not develop only on the basis of a meeting of minds but also—and perhaps primarily and at a deeper implicit level—on recognition and synchronization dynamics, which are, according to the authors, bodily grounded.

Rollo et al. represent the social interactionist standpoint by combining two lines of research: the former on the different ways in which mothers reminisce about shared past experiences with their children, the latter on mothers' use of mental state talk during conversations.

Research indicates that mothers vary in the way in which they reminisce about shared past experiences with their children: “elaborative” or “narrative” mothers show a highly elaborative reminiscing style characterized by long and detailed conversations about the past, interpretation of events and actions in terms of intentions and mental states, frequent references to the autobiographical experiences of their children; “repetitive” or “paradigmatic” mothers, on the other hand, show a less elaborative style, talk less frequently and in less detail about the shared past, focus on labeling objects, and specifying their relations with superordinate categories, or on repeating the exact content of a previous utterance. Children whose mothers are more elaborative during reminiscing seem to develop more sophisticated autobiographical memory skills, such that they tell more detailed and coherent narratives about their personal past.

Besides narrative styles, a large body of research point to the conclusion that the frequency with which mothers use mental state talk (Mental State Language, MSL) during conversations is positively and significantly associated with children's performance in theory-of-mind tasks and the development of their psychological lexicon.

Given this background, the authors sought to put together these two strands of research by examining the relation between narrative styles and the use of MSL in maternal conversations during joint picture-book reading. The results (i) confirmed the validity of the distinction between Narrative and Paradigmatic communicative styles; and indicated that (ii) maternal narrative styles changed as a function of children's age (the mothers of 4-year-old children were more narrative than paradigmatic, whereas the opposite pattern occurred for the mothers of 5- and 6-year-old children); (iii) the use of communicative terms was significantly more frequent for narrative than for paradigmatic mothers, and decreased with children's age; (iv) mothers adapted the referents of their MSL to the children's age (the mothers of younger children referred their MSL more frequently to the book characters, then to themselves or to the child, and lastly to the dyad). Taken together, these results support the idea that mothers adapt their narrative styles and MSL input to the growing abilities of their children—as the latter develop and refine their interaction skills, they move from a narrative to a paradigmatic way of systematizing the reality around them –, thus contributing to the development of sociocognitive understanding.

Veglia and Di Fini explore the universe of personal life-narrations, taken as one of the privileged locus where people try to attribute meanings and look for cohesion and hierarchical order of internal and external information. Their analysis considered different literary genres as well as clinical narrative. Starting from an evolutionary perspective, they postulate the existence of a biological disposition to identify as fundamental six Life Themes: Love, Personal Value, Power, Justice, Truth, and Freedom, laying beyond the apparent, superficial multiplicity of narrations. Interestingly (but not surprisingly, given the evolutionary perspective adopted), a Leitmotif of Life Themes is their relational, interpersonal nature.

The authors delve into the analysis of the role of interpersonal relationships—notably, autobiographical memories related to attachment figures—in personal narrative construction. Most narratives, indeed, are co-created, maintained and revised within the attachment context: parental and domestic relationships during infancy, wider contexts from later childhood on.

While confirming, along with the existent literature, that since childhood secure attachment is related to narrations rich of cooperating caregivers, the authors attest that clinical narrations are characterized by a poor integration due to a rigid or, alternatively, confused exploitation of life themes (“arrested narratives,” “unbounded narratives,” “under narration,” “denarrations”). Starting from this analysis, they suggest that a deeper comprehension of life themes in psychological suffering could help to improve clinical success.

Using the three axes presence/absence of positive or negative narrative occasions, high/low integration, and control of/sharing the theme, the authors identify areas of well-being and, conversely, area of distress, together with important indications concerning the nature of the suffering. This schema, which is co-constructed and shared with the patient and founds a solid therapeutic alliance, gives the therapist a precious tool for envisage and reinterpret together with the patient crucial junctions of her life story, concerning internal, psychological aspects as well as interpersonal relationships.

Beyond its specific, clinical point, the main interest of the paper is its amenability to profitably dialogue and enrich other classical reflections on narrative, such as Dan McAdams' thesis of central nuclei in life stories and John Bruner's perspective on narrative genres.

Rathbone and Moulin introduce the construct of self-image. One component of the Self-Memory-System is the “conceptual self” which is made up of self-images and self-beliefs. Self-images are “I am” statements which are frequently used in psychological research as a measure of identity, as primes in experimental tasks, and as cues to elicit autobiographical memories.

The authors present a descriptive study investigating self-images in adults aged 17 to 88. Participants were presented with an online questionnaire (Study 2) and paper questionnaires/tasks completed in participants' own homes (Study 1) or in the lab (Studies 3, 4, and 5). All participants were asked to generate a set of between 4 and 20 “I am” statements to describe long-term and enduring aspects of their identity. The aim of the paper is to present a database of self-image norms, focusing on people's identity statements.

According to the authors semantic facts about the self may be useful to scaffold narrative continuity and reflect the multi-faceted nature of the self and they are a powerful tool for examining differences in how identity is constructed.

Results revealed that the most commonly generated category of self-image was a social role: Friend followed by Student, Sports player, and Occupation. The less commonly generated category was Articulate and Caring. The highest frequency traits were Happy, Optimistic, and Friendly. Negative self-images (Lazy, Worry, Pessimistic) were also generated multiple times. The youngest self-image category generated was Maths (e.g., “ok at maths”) and the oldest was Serious (e.g., “serious minded”). The most frequently cited self-images for females aged 60–70 was Mother.

Lavazza is concerned with the modulation and potentiation of memory and their relationship with personal identity. In fact, several neuroethicists have recently addressed the use of new possible tools to erase or mitigate negative personal memories. After giving an outline of the state of the art of research in this domain, the author discusses the ethical status of these practices and shows how this can be differently assessed according to different views of the self. In this respect, he takes as being very important the distinction between (partially) normative theories of the self (such as John Bruner's and Marya Schechtman's narrative theories), and purely naturalistic theories of the self (e.g., Peter Carruthers).

The artificial alteration of identity will be taken ethically unacceptable only on those conceptions and models of personal identity and self-according to which these act as reference points also for a wider set of values. For instance, some authors complained that chemical alteration of one's memory might lead to non-authentic choices. The idea is that the choice must be in line with the “true” self, to the extent that this latter notion makes sense.

Narrativist theories are inclined to put in question the idea of a strong self, in virtue of their insistence of the continuously tentative character of personal identity. Narratives write and rewrite life stories incessantly. By contrast, neurobiological theories of the self-promote a strong idea of the self, for they identify the psychic healthy state in a neurobiological condition. Use of drugs aims at maintaining this condition. Hence, Lavazza seems point here to a contradiction: use of molecules to modulate memories is ethically (more) allowed if one endorses a weak notion of the self, but neurobiological approaches to the self are committed to a strong notion of the self.

## Trauma and Disruption Of Autobiographical Memory

Hart et al. aim at assessing the relationship between neuropsychological functioning (notably, working memory and cognitive flexibility), Post-traumatic Stress Disorders (PTSD), and trauma centrality, i.e., the extent to which a traumatic event is taken as central to one's identity and a turning point in the story of our life. To this empirical research based on a cross-sectional design took place 41 Iran and Afghanistan veterans. Although some features of the study (small and full masculine sample, use of on-line neuropsychological test) suggest further investigation, a correlation emerges. While a growing body of empirical research has already well-established the association between, on the one side, PTSD symptoms and trauma centrality, and, on the other side, neuropsychological disorders and PTSD, the possible correlation between neuropsychological functions and trauma centrality is the main focus of this study.

Beyond replicating the solid data attesting the other correlations, the present research shows that lower levels of working memory and (even if results only approach significance) cognitive flexibility are associated with trauma centrality (ceteris paribus, i.e., when measures of depression and PTSD are controlled). However, the results, which are coherent with the well-known overgeneralization of memories in traumatized people, cannot shed light on the direction-of-causality question, as cross-sectional studies assess all the variables at the same time.

Typically, cognitive deficits are unidirectionally taken to affect trauma centrality and, more generally, identity; but things could be more complex and interwoven, as suggested by Conway's work. In his Self-Memory System Conway postulates a strict, continuous and bidirectional relationship between long term mnemonic structures and the working self, i.e., the hierarchical structure containing currently active personal goals. In this theoretical framework, deficits in working representations affect long term self-structures, conceived both as episodic memories and normative, semantic self-structures. Working self constantly mediates between the opposite requirements of internal coherence and correspondence to reality, allowing only the “right kind” of new acquired information to be stored in long-term memory. SMS postulates also the opposite causal direction, suggesting that permanent structures of the self-influence current decisions taken by the working self. As well shown in Conway et al. (2004), structures of personality—in their Bowlbian perspective, internal working models—not only are modulated by, but also modulate working self's functioning.

Carletto et al. view Post-Traumatic Stress Disorders (PTSD) as the crucial disorder to investigate autobiographical memory. They present Eye Movement Desensitization and Reprocessing (EMDR) as the therapeutic technique able to re-elaborate non-integrated memories and to consolidate new memories into the already existing semantic links, promoting the insertion of traumatic but no longer disturbing memory in a coherent and adaptive autobiography.

PTSD is a disorder that develops in some people who have experienced a shocking, scary, or dangerous event. People who have PTSD may feel stressed or frightened even when they are not in danger. The diagnosis include at least one re-experiencing symptom (e.g., flashbacks—reliving the trauma over and over, including physical symptoms like a racing heart or sweating, bad dreams, frightening thoughts), at least one avoidance symptom (e.g. staying away from places, events, or objects that are reminders of the traumatic experience, avoiding thoughts or feelings related to the traumatic event), at least two arousal and reactivity symptoms (e.g., being easily startled, feeling tense or “on edge,” having difficulty sleeping, having angry outbursts), at least two cognition and mood symptoms (e.g. trouble remembering key features of the traumatic event, negative thoughts about oneself or the world, distorted feelings like guilt or blame, loss of interest in enjoyable activities).

Traumatic memories are encoded differently than memories of ordinary events, in multisensory fragments that traumatized people cannot integrate in a structured narrative. From a neurobiological point of view, during extreme stressful situation amygdalar synapses are potentiated and this saturate all amygdalar alpha-amino-3-hydroxy-5-methyl-4-isoxazole (AMPA) receptors-bindings sites. Consequently, in the hippocampus the recorded emotional memory trace cannot be merged with the cognitive memory trace and the fragments of emotionally charged memories remain trapped in the limbic system and cannot be transferred to the cortical areas, where a further processing and integration into already existing networks should take place. Therefore, non-processed emotional memories remain trapped and unchanged at subcortical level without contextual integration, causing in some cases PTSD symptoms.

If memories are adequately processed, PTSD symptoms can be mitigated and memories integrated. EMDR is the psychological treatment for PTSD presented by authors. The key aspect is the use of Alternate Bilateral Stimulations (ABS) such as eye movements (EMs).

Authors describe how EMDR works during the PTSD treatment from a neurobiological point of view, mentioning the same mechanism involved in sleep for memory consolidation, arguing also for a potential role for cerebellum in the processes involved in trauma.

## Autobiographical Memory and Psychotherapeutic Versus Pharmacological Treatments

Mancini and Mancini describe ImRs (“Imagery Rescripting”), a therapeutic technique that aims to reduce the distress associated with negative memories of early aversive experiences. It consists of prompting patients to rescript the autobiographical memory in line with their unmet needs.

The ImRs procedure is based on two steps: (i) change in perspective, from child's one to adult's one; (ii) try to meet the child's unmet needs. The second step requires empathy: empathy on behalf of the adult validates the child's suffering. This makes explicit the rewriting work: the (negative) meaning inferred during childhood (e.g., in attachment relations) is overturned in the recognition of the child's suffering.

ImRs has proven to be effective across several psychopathologies such as depression, social phobia, obsessive-compulsive disorder, post-traumatic stress disorder, and personality disorders. Yet, the mechanisms through the result is obtained are not properly understood. The hypothesis of the authors is that ImRs acts by reducing the meta-emotional problem, i.e., by reducing the perception of a negative emotion as something problematic and unacceptable. A large literature mentioned by the authors seems to show that the meta-emotional problem could be a trans-diagnostic phenomenon. Yet, since there is still a lack of direct evidence that ImRs reduces the meta-emotional problem, the authors propose an experiment to test their hypothesis.

In sum, the authors argue that ImRs promotes the reduction of symptoms and of negative self-belief both by changing the valence of the aversive autobiographical memory and by reducing their accessibility—both approaches offer a plausible explanation of the observed reduction in negative self-belief. However, they also believe that these approaches are not very accurate in clarifying how the change in self-belief occurs. They suggest establishing a link between the change occurring in the beliefs about one's own emotion and the change occurring in self-belief.

Kalsi et al. present the first neuroimaging meta-analysis which report the changes in functional neural activity as an effect of pre- and post-treatment of psychotherapy versus pharmacological therapy in patients with affective disorders (anxiety and depression).

The main outcome is the inverse effects of cognitive and psychodynamic psychotherapy and antidepressant therapy on the right paracingulate activity. The patients undergoing psychotherapy showed an increase in the right paracingulate activity while pharmacological treatment led to a decrease of activation of this area. The authors conclude that this finding seems to support the recent studies that hypothesize how psychotherapy, through the self-knowledge and the meaning processing, involves a top-down emotional regulation.

What is most remarkable for the Research Topic is that the paracingulate cortex activity seems to be associated with autobiographical memory—as well as with other self-monitoring activities (visual self-recognition, conflict monitoring, verbal self-monitoring, self-generated thoughts) and mentalizing abilities.

## Conclusion

The main goal of this Research Topic “Self and Memory: A Multidisciplinary Debate” was to offer a snapshot of the growing cognitive, developmental, and clinical psychological literature on the complex relationship between autobiographical memory and the self against the background of the main theoretical approaches in this research area. The final collection of 12 articles does exactly that and provide hints about where this research area is going.

The contributions have all a multidisciplinary character, but some of them focus more on one of the three strands of thinking around self and memory described above (line 17), others focus mostly on issues in the blossoming empirical literature of psychology, psychopathology, and neuroscience of self and memory. More specifically, Tippett et al., Wheeler and Gabbert, and Rathbone and Moulin's papers make the most of Tulving's approach to autobiographical memory; Vanderveren et al., Veglia and Di Fini, and Hart et al.'s contributions build on Conway's perspective; Marchetti et al., Rollo et al., and Lavazza take advantage of Nelson's approach to autobiographical memory. Finally, the contributions of Carletto et al., Mancini and Mancini, and Kalsi et al. focus on clinical issues that cross-cut the three theoretical perspectives—EMDR and Post-Traumatic Stress Disorders; the influence of Imagery Rescripting on the aversive autobiographical memory; neuroimaging data about the effects of pre- and post-treatment of psychotherapy vs. pharmacological therapy.

We hope that the reader will find the collected articles both informative and thought-provoking, and that this Research Topic will serve to stimulate new theoretical debate and empirical research.

## Author Contributions

All authors listed have made a substantial, direct and intellectual contribution to the work, and approved it for publication.

### Conflict of Interest Statement

The authors declare that the research was conducted in the absence of any commercial or financial relationships that could be construed as a potential conflict of interest.
